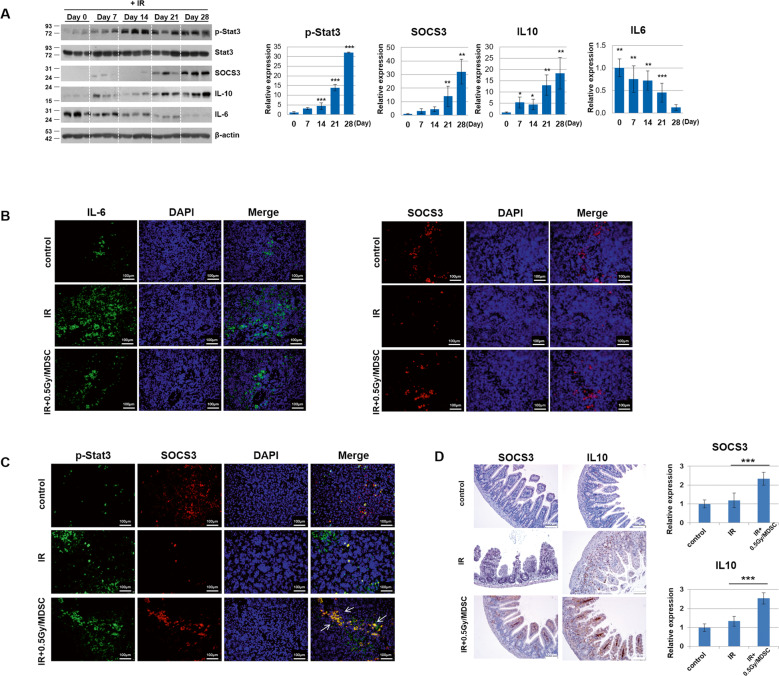# Correction: Expansion of monocytic myeloid-derived suppressor cells ameliorated intestinal inflammatory response by radiation through SOCS3 expression

**DOI:** 10.1038/s41419-022-04643-w

**Published:** 2022-02-25

**Authors:** You Yeon Choi, Ki Moon Seong, Hyun Jung Lee, Seung Sook Lee, Areumnuri Kim

**Affiliations:** 1 Laboratory of Biodosimetry, National Radiation Emergency Medical Center, KIRAMS, Seoul, 01812 Korea; 2 Laboratory of Radiation Exposure and Therapeutics, National Radiation Emergency Medical Center, KIRAMS, Seoul, 01812 Korea; 3 Department of Pathology, Korea Cancer Center Hospital, Korea Institute of Radiological & Medical Science, Seoul, 01812 Korea

**Keywords:** Stress signalling, Super-resolution microscopy

Correction to: *Cell Death and Disease* 10.1038/s41419-021-04103-x, published online 03 September 2021

The original version of this article unfortunately contained an error in figure 5d. The authors apologize for the error. The amended figure can be found below. The original article has been corrected.